# Local Regulatory T-cell Failure as a Potential Mechanism of the Halo Sign in Spinal Instrumentation: A Mechanistic Hypothesis

**DOI:** 10.7759/cureus.114200

**Published:** 2026-08-09

**Authors:** Mehmet Faik Ozveren

**Affiliations:** 1 Neurosurgery, Kutahya Anadolu Hospital, Kutahya, TUR

**Keywords:** bone marrow, immunological consequences, osteoclastogenesis, pedicle screw, regulatory t-lymphocytes

## Abstract

The radiolucent halo seen on computed tomography (CT) around a loosening pedicle screw is usually interpreted as a mechanical sign, explained by micromotion, wear debris, and particle disease. Those explanations come from hip and knee arthroplasty. A pedicle screw is not an arthroplasty component; it is driven into the vertebral body, so part of its surface lies within the bone marrow from the moment of insertion.

This article asks whether that difference has immunological consequences. The proposal is that metal-derived products may be presented by bone marrow antigen-presenting cells (APCs) to local T cells, supporting an in situ type IV immune response. That response may stay clinically silent while local regulation holds. Loss of that regulation may then permit receptor activator of nuclear factor-κB ligand (RANKL)-driven osteoclastogenesis along the screw. The provisional term “Sakaguchi-type regulatory failure” is used for that proposed local loss of regulatory T-cell (Treg)-mediated restraint. It is not a recognized pathological or radiological entity.

If the model has merit, patients whose regulatory capacity is already impaired should show more implant-related failure, and the spinal literature is compatible with that. The reported excess, however, is in reoperation rather than the halo sign and is confounded by bone density, corticosteroid use, and low-grade infection. Spine-specific evidence further constrains the model, and the three findings that do so are set out alongside the case for it. No direct human spinal evidence exists either in support of or against the hypothesis. Predictions that would refute the hypothesis are also presented. This is a hypothesis article, not a systematic review.

## Introduction and background

The established mechanical explanation

Pedicle screw loosening is common after instrumented fusion. The conventional explanation is mechanical. Repetitive micromotion, the toggling or windshield-wiper effect, grinds the surrounding bone and drives pressure- and debris-mediated osteolysis. Poor bone quality lowers the threshold at which this occurs [1,2]. On this view, the radiolucent zone reflects a failing bone-implant interface, and the evidence supporting this interpretation is strong [3,4]. Nothing in this article disputes that.

Current evidence on implant-associated immune reactions

A second body of literature describes immune reactions to orthopedic implants, almost all of it from the hip and the knee. There, the prosthesis moves inside a synovial capsule and sheds submicron wear particles, which macrophages carry to regional lymph nodes; the T-cell response is therefore primed at a distance from the implant [5]. Metal hypersensitivity is recognized in that setting and has a diagnostic framework [6,7]. Three things are often run together and should be separated: delayed-type hypersensitivity to metal-derived antigens, the innate foreign-body reaction to particulate debris, and classical organ-specific autoimmunity against native antigens. This article concerns the first. It borrows an experimental idea from the third. It does not claim that peri-screw osteolysis is an autoimmune disease.

The gap in the spinal literature

A pedicle screw does not articulate. It is passed through the pedicle into cancellous bone, and once seated, its surface stays in contact with marrow. Marrow contains T cells, antigen-presenting cells (APCs), and cluster of differentiation 4 and 25 positive (CD4+CD25+) Tregs and has been described as capable of behaving in some respects as a secondary lymphoid organ [8,9]. Metal ions are released from spinal instrumentation, with local intraoperative concentrations exceeding serum concentrations, although the measurements come from pediatric deformity surgery rather than adult degenerative fusion [10,11]. Yet no study has characterized the peri-screw infiltrate by immunophenotype, and none has examined marrow Treg number or function at an instrumented vertebra. The spinal literature has concentrated on wear-debris biology and material behavior [12].

The proposed hypothesis

The proposal is that metal-derived ions or metal-modified host proteins may be processed and presented by local marrow APCs to resident or recruited T cells, potentially supporting an in situ type IV response. The relevant subtypes would be IVa and IVc, as in metal contact hypersensitivity. Antigen recognition may remain clinically silent when regulatory mechanisms and local tissue homeostasis are preserved. Loss of local immune regulation may permit an exaggerated antigen-specific inflammatory response. The manuscript examines whether an analogous regulatory failure could contribute to peri-implant bone loss. The analogy is to Sakaguchi and colleagues, who showed that removing one regulatory T-cell (Treg) subset from a normal mouse was enough to unleash organ-specific autoimmune destruction [13,14].

For the purposes of this hypothesis, we use the provisional term “Sakaguchi-type regulatory failure” to describe the proposed local loss of Treg-mediated restraint. It is a descriptive label. It is not a recognized pathological or radiological entity and has no diagnostic criteria. Three levels of claim are kept separate throughout: what is established, what is extrapolated from arthroplasty or animal work, and what is speculation specific to the spine. The data below assign each chain link to one of these classes in tabular form.

## Review

Methods of narrative literature selection

This article is a hypothesis and narrative opinion piece. It is not a systematic review. No protocol was registered. No formal eligibility criteria were applied. Screening was not duplicated. No risk-of-bias or publication-bias assessment was performed, and no quantitative synthesis was conducted.

PubMed/MEDLINE and Google Scholar were searched from inception to July 2026 for English-language publications. Terms were combined from the following: pedicle screw loosening, radiolucent zone, halo sign, peri-implant osteolysis, periprosthetic osteolysis, metal hypersensitivity, haptenation, metal ion release, bone marrow immunity, Treg, Foxp3, CD4+CD25+, RANKL, osteoprotegerin (OPG), rheumatoid arthritis spine surgery, and implant-associated infection. Reference lists of retrieved articles were screened by hand. Priority was given to primary experimental work, prospective clinical series using an objective standard for loosening, and findings that contradict the model.

The selection is illustrative rather than exhaustive, and it carries the bias that follows from that: evidence consistent with a proposed mechanism is easier to find when one is looking for it. Contrary findings known to us are cited and addressed rather than passed over. The reference list should be read as an argument, not a survey.

Clinical and radiological definition of the peri-screw halo

Peri-screw loosening is commonly assessed as a radiolucent zone of approximately 1 mm or greater on radiography or computed tomography (CT), although thresholds, required circumferential extent, and imaging accuracy vary between studies [3,4,15]. The zone is best seen on CT bone windows. It usually becomes apparent months rather than days after surgery.

Three series have tested peri-screw lucency against extraction torque measured at screw removal, and the imaging modality differs between them. On dedicated oblique radiographs, the sign was 100% specific and 64% sensitive across 79 screws [3]. On CT, it was 96% specific and 22% sensitive in the second series, in which plain radiography had 24% sensitivity, and 96.7% specific and 64.8% sensitive in the third, in which radiography had 54.2% sensitivity and 83.5% specificity [4,15]. Specificity is therefore consistently high; sensitivity is not. Technique explains part of the spread, since oblique projections reveal zones that standard views miss and metal artifact degrades the peri-implant image [3,4]. The torque threshold used to define a loose screw explains more: 40 N·cm in the first series, 60 N·cm in the third, and two and a half times the first in the second [3,4,15]. A higher threshold counts mildly loose screws as loose, and sensitivity falls accordingly.

What remains is an asymmetry. A halo usually means a loose screw, but a screw can be loose without one [3,4,15]. The halo is therefore not a synonym for loosening; it marks a subset of it, and that subset is the lesion this hypothesis concerns: a late, progressive, circumferential lucency that widens over months. A faint early peri-screw lucency may occasionally reflect postoperative remodeling; however, its frequency and clinical significance require supporting evidence.

One gap should be noted because the hypothesis inherits it. Observer-variability data exist for postoperative CT of transpedicular instrumentation, but they concern screw position rather than the radiolucent zone [16]. We are aware of no published interobserver reliability data for the halo itself, and the two series that first validated the sign each used a single reader [3,4]. A hypothesis resting on a radiological marker is only as reproducible as that marker. One remedy is to stop relying on a binary reading: peri-screw attenuation in Hounsfield units is continuous and quantitative, is obtainable from the same scan, and is proposed below as a secondary endpoint. It is not free of observer effects: region-of-interest placement, reconstruction parameters, and metal artifact all affect it and would have to be standardized before it could be compared across centers.

Established mechanisms: mechanical, particle-mediated, and infectious

The mechanical account is not in doubt. Screws surrounded by a radiolucent zone have far lower mean extraction torque than well-fixed screws, 16±10 N·cm versus 403±220 N·cm. In a sheep model, the same screws show lower pull-out resistance (243±156 versus 2214±578 N) and a much smaller bone-to-screw contact area (8±9% versus 55±29%) [3]. Low bone density sits near the top of every list of risk factors [1,2,17].

The particle-mediated account comes from arthroplasty, where wear debris drives macrophage activation and periprosthetic osteolysis [5,12]. A pedicle screw has no articulating bearing, so the particulate burden differs, but ion release is measurable, as noted above. Two qualifications belong here. The spinal measurements were made in children undergoing instrumented correction for scoliosis, and the exposure in adults undergoing degenerative fusion has not been quantified [10,11]. Corrosion in a modular construct is also not confined to the bone-screw interface. The rod-screw junction is a recognized site of fretting and crevice corrosion, and debris generated there is shed into paraspinal soft tissue rather than into marrow. How much of the ion load actually reaches the vertebral body is unknown. The exposure this hypothesis requires is therefore plausible rather than established.

The infectious account has strengthened and must be taken seriously. Sonication of pedicle screws removed from patients with clinical loosening but no signs of infection recovered low-virulence organisms in 40.7% of cases, and in none of the non-loosened controls [18]. That figure makes indolent biofilm-associated infection the leading competing explanation for the lesion described here. Any immunological account has to be distinguished from it before it can be entertained. We return to this in the Limitations section.

Bone marrow immunity and metal hypersensitivity

Titanium and its alloys are often called inert. At the molecular level, they are not. Biotribocorrosion at the screw-bone interface releases titanium, aluminum, and vanadium, and, in some alloys, nickel, cobalt, or chromium. The proposed first hit is exposure of marrow to metal-derived products, the magnitude of which likely varies with alloy composition, corrosion, surface treatment, and micromotion [11,12]. Metal ions may bind to or modify host proteins, potentially generating antigenic metal-protein complexes capable of immune recognition [7].

Whether such complexes are presented locally is the point at issue. Marrow contains APCs and both effector and Treg populations, and Ort and colleagues have argued that it can mount and sustain hypersensitivity responses to implant-derived material in situ [8]. Peri-implant lymphocytes from loose implants are more activated and proliferate more strongly than those from stable ones, although those data come from arthroplasty [19]. A local elicitation response may occur within marrow, although initial sensitization or amplification through regional lymphoid tissue cannot be excluded. The model does not require marrow to prime naive cells. Marrow T cells are predominantly memory cells, and marrow is better described as a site where memory populations are maintained and reactivated. A pre-existing pool of metal-reactive memory cells, sensitized earlier by cutaneous, dietary, occupational, or previous implant exposure and reactivated at the screw surface, may facilitate the proposed mechanism; local priming, or a contribution from regional lymphoid tissue, cannot be excluded. Prior metal exposure therefore becomes a measurable covariate rather than a silent assumption.

One objection to a marrow-based account is geographical: how would regulatory cells come to be at the implant surface? Bone marrow is itself a reservoir for CD4+CD25+ Tregs, which traffic to it and are retained through the C-X-C motif chemokine ligand 12 (CXCL12)-C-X-C motif chemokine receptor 4 (CXCR4) axis [9]. The cells the hypothesis needs are plausibly already resident.

A second objection is compositional, and it is the harder of the two. Vertebral marrow is not uniformly hematopoietic. Red marrow converts to fat with age, and marrow fat rises further as bone density falls; in postmenopausal women, vertebral marrow proton density fat fraction is inversely related to bone mineral density [20]. The patients who experience loosening most are therefore the patients whose vertebral marrow is most fatty and least lymphocyte-rich. Vertebral bodies retain hematopoietic marrow longer than the appendicular skeleton, and fat-replaced marrow is not devoid of lymphocytes, so this does not dispose of the model. It does mean the cellular substrate should be demonstrated rather than assumed and that marrow fat fraction, which chemical shift MRI measures noninvasively, belongs in any test of the hypothesis.

Metal ions may alter lymphocyte proliferation and cytokine signaling; however, whether this selectively impairs local Treg survival or IL-2 availability at the screw interface remains unknown. Titanium, cobalt, and chromium ions inhibit T-cell proliferation in vitro, and interleukin-2 reverses the effect [21]. Tregs depend heavily on interleukin-2 for survival. A local fall in available interleukin-2 could therefore weaken the regulatory compartment before it weakens the effectors. The same data support the opposite reading. Metal ions inhibited both T- and B-cell proliferation, so broad local suppression is at least as compatible with immunological quiescence at a metal surface as with selective regulatory failure. Measuring suppressive function rather than proliferation would settle it. Both readings are inferences from in vitro work, and neither has been tested in bone.

The Sakaguchi paradigm and what it does and does not license

In 1985, Sakaguchi and co-workers removed one T-cell subset from otherwise normal mice and produced organ-specific autoimmune disease. Restoring the cells prevented it [13]. A decade later, the subset was identified as carrying CD4 together with CD25, and the depletion-and-reconstitution result was reproduced in a dose- and timing-dependent manner [14]. Foxp3 was later identified as the transcription factor that programs it [22,23].

Two features of that work support the present hypothesis. Self-reactive effector cells are already present in normal tissue; they need not be introduced, only released. Regulatory cells also act dominantly, raising the threshold at which neighboring effectors are activated.

Two caveats. Murine CD25 biology does not map cleanly onto human Foxp3-defined Tregs [22,23]. The analogy drawn here is to the mechanism of regulatory failure, not to a claim that peri-screw osteolysis is a classical autoimmune disease.

The proposed regulatory failure mechanism

The proposal is a two-step sequence. The first hit is the exposure described above. The proposed second hit is erosion of local regulatory capacity, whether from a continuous antigen load, immunosenescence, or a systemic autoimmune disease the patient already carries. A visible halo would require both. It should therefore appear soonest and most severely where regulatory capacity was weakest to begin with.

If regulatory restraint were lost, the local cytokine balance would be expected to shift. Th17-derived interleukin-17 induces receptor activator of nuclear factor-κB ligand (RANKL) on osteoblasts and stromal cells, and effector T cells can express RANKL directly. Tumor necrosis factor-α (TNF-α) and interleukin-17 (IL-17) can promote osteoclastogenic signaling, whereas interferon-γ has context-dependent effects and may either inhibit RANKL-mediated osteoclast differentiation or contribute indirectly to inflammation [24]. What determines whether resorption proceeds is the RANKL-to-OPG ratio at the interface, not RANKL alone [25]. Tregs restrain osteoclastogenesis by cell contact and through interleukin-4 and interleukin-10, so losing regulatory output would remove active suppression rather than merely permit resorption [26].

**Figure 1:**
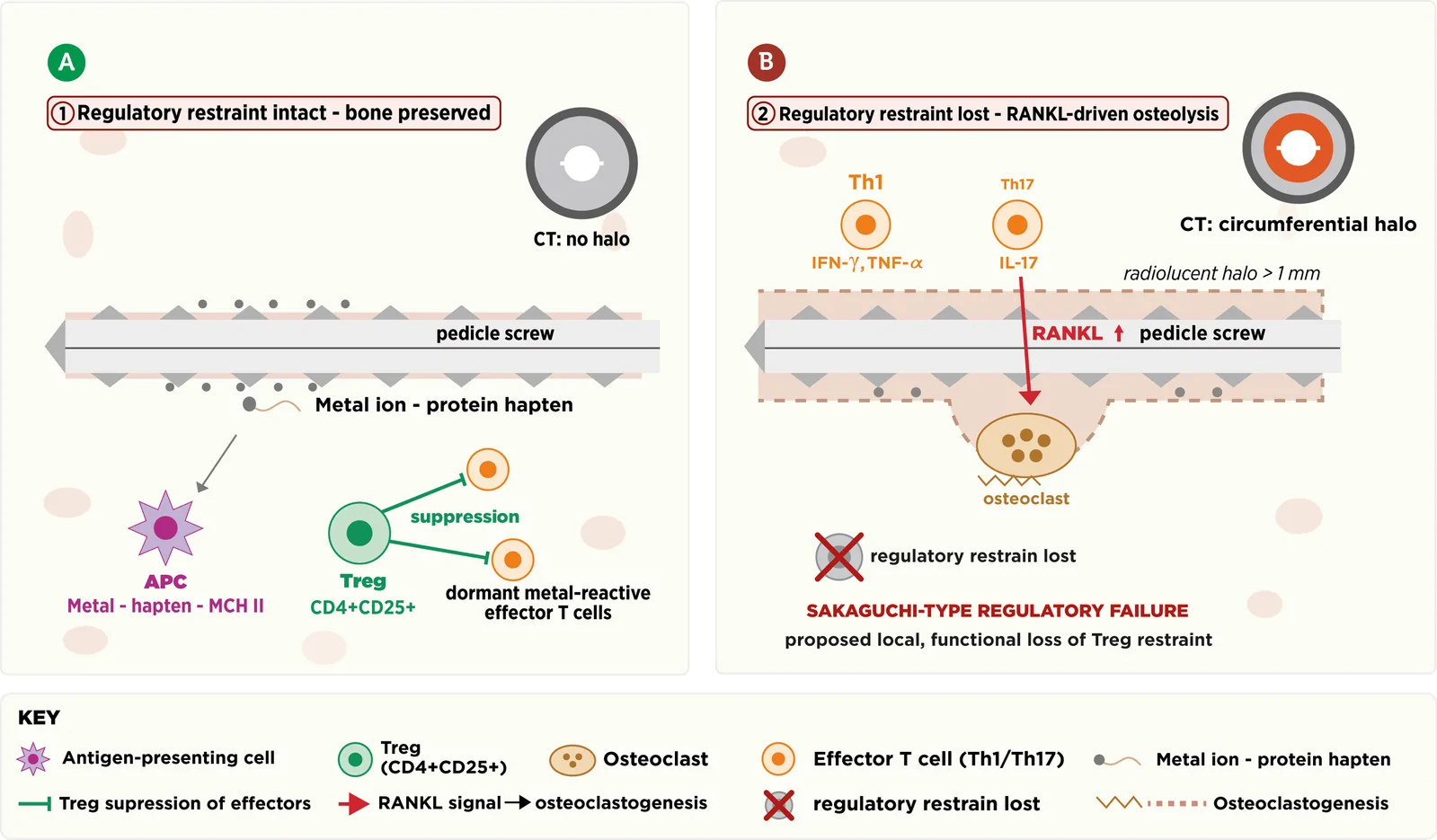
Proposed sequence at the screw-marrow interface under the local regulatory failure model (A) Regulatory restraint intact. The pedicle screw surface contacts vertebral marrow, where released metal ions may bind host proteins and be presented by APCs to CD4+ T cells. When CD4+CD25+ Treg restrain metal-reactive effector cells, these cells remain dormant, bone is preserved, and CT shows no peri-screw halo. (B) Regulatory restraint lost. If local regulatory control fails, Th1 and Th17 effectors may be released. IFN-γ, TNF-α, and IL-17 are proposed to increase the RANKL/OPG ratio at the interface, promoting osteoclastogenesis. Osteoclast-mediated resorption along the screw contour produces the circumferential radiolucent zone wider than 1 mm seen on CT. The key identifies the cells, signals and symbols used in both panels. “Sakaguchi-type regulatory failure” is used here as a provisional descriptive term. It is not a recognised pathological or radiological entity and has no diagnostic criteria. The figure presents a mechanistic hypothesis, not demonstrated in situ metal recognition or Treg failure in human vertebral marrow. APCs: antigen-presenting cells; CT: computed tomography; IFN-γ: interferon-γ; IL-17: interleukin-17; MHC: major histocompatibility complex; RANKL: receptor activator of nuclear factor-κB ligand; TNF-α: tumour necrosis factor-α; Th: T-helper; Treg: regulatory T-cell; OPG: osteoprotegerin The figure was created using CorelDRAW (Corel Corporation, Ottawa, ON, Canada).

Resorption in this model would occur where the antigen is recognized, which is along the implant surface. Bone would be lost as a shell following the screw contour. Table 1 sets out the sequence, and Figure 1 illustrates it. Table 2 states what class of evidence supports each link.

**Table 1:** Proposed stepwise sequence of halo sign formation under the local regulatory failure model, with the level of evidence for each step Citations denote the published literature supporting each component step. They document the established biology on which the model draws and are not a claim that the hypothesized sequence has itself been demonstrated in the instrumented spine. Evidence level is graded as established (demonstrated in relevant human or animal tissue), indirectly supported (demonstrated in a related setting, chiefly arthroplasty or in vitro), or speculative (not demonstrated in any setting relevant to the spinal metal-marrow interface). CD4+CD25+: cluster of differentiation 4 and 25 positive; IL-17: interleukin-17; OPG: osteoprotegerin; RANK: receptor activator of nuclear factor-κB; RANKL: RANK ligand; Th17: T-helper 17; TNF-α: tumor necrosis factor-α; Treg: regulatory T-cell; Ti: titanium; Co: cobalt; Cr: chromium; APCs: antigen-presenting cells; CT: computed tomography; MHC: major histocompatibility complex; Th1: T-helper 1; TRAF6: TNF receptor-associated factor 6; IFN-γ: interferon-γ; Al: aluminum; V: vanadium; Ni: nickel; NFATc1: nuclear factor of activated T cells

Step	Event at the screw-marrow interface	Proposed immunological event	Radiological/clinical read-out	Evidence level
1	Screw embedded in the cancellous vertebral body; bare metal surface in direct, continuous contact with bone marrow from implantation [8]	Resident T cells, APCs and CD4+CD25+ Tregs share the implant microspace; baseline self-tolerance is maintained by local regulatory restraint [8,9,13]	Immediate postoperative period: no halo; osseointegration proceeds normally [3]	Established. Marrow immune composition and direct screw-marrow contact are demonstrated; immunological quiescence is inferred
2	Biotribocorrosion releases metal ions (Ti, Al, V; ± Ni, Co, Cr), which may coordinate to host proteins and potentially form hapten-protein neoantigens [7,10]	Proposed event: marrow APCs may process metal-modified proteins and present relevant antigenic complexes, displayed on MHC II, to local or recruited CD4+ T cells, potentially supporting an in situ type IV response [7,8]	Subclinical; detectable only as low-grade local T-cell activation. Halo not yet visible [19]	Indirectly supported. Ion release from spinal implants is demonstrated; haptenation and lymphocyte activation are shown in arthroplasty and in vitro; in situ presentation at the spinal screw surface has not
3	Continuous antigen load plus host factors (immunosenescence, systemic autoimmune disease, limited-course immunosuppression) may erode local regulatory capacity [14,27]	Proposed functional equivalent of CD25+ Treg depletion: metal-reactive effector cells released, Th1/Th17 dominance emerges (Sakaguchi-type regulatory failure) [13,14]	Early radiolucency may appear, potentially earlier where regulatory capacity is impaired; recurrent pain in a previously improving patient [1]	Speculative. No data describe marrow Treg number or function at an instrumented vertebra; the analogous defect is documented in rheumatoid marrow at hip replacement only
4	RANKL signaling rises relative to OPG at the interface; osteoclasts resorb bone along the screw contour [25]	Th17-derived IL-17 induces RANKL on osteoblasts and stromal cells; effector T cells express RANKL directly. TNF-α and IL-17 can promote osteoclastogenic signaling, whereas IFN-γ has context-dependent effects and may either inhibit RANKL-mediated osteoclast differentiation or contribute indirectly to inflammation. Signaling through RANKL-RANK-TRAF6 then converges on NFATc1 and drives the maturation of bone-resorbing osteoclasts [25]	Circumferential radiolucent halo >1 mm on CT; progressive loosening; risk of pseudarthrosis, construct failure, deformity recurrence [3,4,15]	Established biology, speculative application. The RANKL/OPG axis and its regulation by T-cell cytokines are established; whether it is driven by regulatory failure at the peri-screw interface remains untested

**Table 2:** Provenance of the evidence supporting each mechanistic link in the proposed model Evidence class indicates the setting in which the link has been demonstrated, not the strength of the individual studies. Direct spinal: demonstrated in instrumented human or animal spinal tissue. Arthroplasty: demonstrated in hip or knee periprosthetic tissue. Animal/in vitro: demonstrated in experimental systems not specific to implants. Extrapolation: not demonstrated in any of the above settings and asserted by analogy. Where two settings are given, the link has been demonstrated in both; “confounded” indicates that the cited evidence cannot be attributed to the proposed mechanism because an alternative explanation was not controlled for. CD4+CD25+: cluster of differentiation 4 and 25 positive; CXCL12: C-X-C motif chemokine ligand 12; CXCR4: C-X-C motif chemokine receptor 4; IL-17: interleukin-17; OPG: osteoprotegerin; RANKL: receptor activator of nuclear factor-κB ligand; Th17: T-helper 17; TNF-α: tumor necrosis factor-α; Treg: regulatory T-cell; Ti: titanium; Co: cobalt; Cr: chromium.

Mechanistic link	Strongest available evidence	Evidence class	References
Part of the pedicle screw surface lies in direct, continuous contact with the vertebral marrow	Anatomical; not contested	Direct spinal	[8]
Metal ions are released from spinal instrumentation, with local concentrations exceeding systemic ones	Prospective measurement of local and serum metal ion levels in instrumented patients	Direct spinal	[10,11]
Metal ions bind or modify host proteins to form complexes capable of immune recognition	Arthroplasty hypersensitivity literature and in vitro work	Arthroplasty/in vitro	[7]
Bone marrow can act as a site of antigen presentation and sustain hypersensitivity responses	Review of human marrow immuno-capacity in metal-associated hypersensitivity	Arthroplasty/human marrow	[8]
Peri-implant lymphocytes from loose implants are more activated than those from stable implants	Comparative analysis of periprosthetic tissue	Arthroplasty	[19]
CD4+CD25+ Tregs reside in bone marrow and are retained via CXCL12-CXCR4	Human and murine trafficking studies	Animal/in vitro	[9]
Metal ions impair lymphocyte proliferation in an interleukin-2-reversible manner	In vitro exposure of T and B cells to Ti, Co, and Cr	In vitro	[21]
Depletion of a Treg subset alone is sufficient to cause organ-specific autoimmunity	Murine depletion-and-reconstitution experiments	Animal	[13,14]
Th17/IL-17 and TNF-α raise the RANKL-to-OPG ratio; Tregs restrain osteoclastogenesis	Osteoimmunology of bone turnover and periprosthetic osteolysis	Animal/in vitro	[24-26]
Marrow Tregs are reduced in number and functionally impaired in rheumatoid patients	Direct marrow sampling at hip replacement, rheumatoid versus osteoarthritic controls	Human marrow, arthroplasty setting	[27]
Patients with rheumatoid disease loosen and require reoperation more often	Systematic review and meta-analysis. Confounded by bone density and corticosteroid exposure	Direct spinal, clinical, and confounded	[28]
Hydroxyapatite coating reduces peri-screw radiolucency by limiting antigen exposure	Coated versus uncoated screws in a single series; cannot be separated from improved osseointegration	Direct spinal and confounded	[3]
Local regulatory failure at the metal-marrow interface causes the peri-screw halo	No supporting or refuting data in any setting; this is the hypothesis itself	Extrapolation	-

Evidence supporting the hypothesis

In the series that defined the radiolucent zone, 13 of 21 patients received plasma-sprayed hydroxyapatite-coated screws. Radiolucent zones developed around 53% of the uncoated screws and 2% of the coated ones [3]. The coating is some 45 µm of ceramic and does not alter the thread, geometry, or loading. Hydroxyapatite coating both separates part of the metal surface from direct marrow contact and enhances osseointegration. Consequently, the reported reduction in radiolucency cannot distinguish an immunological barrier effect from improved mechanical fixation. Covering the metal surface may reduce exposure of local APCs to metal-derived products, although it may not eliminate such exposure. The observation is compatible with the hypothesis. It does not test it. The two readings diverge in what they predict, and Prediction 7 exploits that divergence. Two further features of that series count against the model rather than for it and are taken up in the next section.

If a halo required both a universal exposure and a variable regulatory failure, patients with impaired regulatory capacity should experience loosening more often. Rheumatoid arthritis is characterized by impaired Treg function [27]. That is the group in which the model predicts the most halo. The spine literature, written with none of this in mind, points the same way. A pooled analysis reports that infection, implant-related complications, and reoperation were increased roughly two- to fourfold in patients with rheumatoid arthritis [28]. Several outcome studies exclude patients with rheumatoid arthritis outright. Exclusion of patients with rheumatoid arthritis from some studies reflects their distinct clinical risk profile but does not, by itself, identify the mechanism responsible for poorer outcomes.

The nearest direct evidence is not spinal. Massalska and colleagues sampled marrow at hip replacement. They found significantly fewer CD4+ forkhead box P3-positive (FOXP3+) Tregs in patients with rheumatoid arthritis than in those with osteoarthritis, and the remaining cells were functionally impaired, producing tumor necrosis factor while still attempting to suppress [27]. That is a defective regulatory compartment in human marrow. It is not one at an instrumented vertebra. The qualification in that paper's own title should also be carried over: marrow regulatory cells from the osteoarthritic controls suppressed poorly as well, so the defect described is partial and may not be specific to rheumatoid arthritis.

Evidence and arguments against the hypothesis

Three spine-specific observations challenge this model, and none of them is borrowed from arthroplasty. The first is prevalence. In a consecutive pediatric series of 796 patients undergoing spinal instrumentation, 118 screened positive on a history questionnaire, but patch testing confirmed metal hypersensitivity in only 26, about 3% of the cohort, with nickel being the most common allergen at 2.5% [29]. A systematic review of six studies and 806 participants arrived at the same figure of roughly 3% in pediatric populations, again with nickel being the most common allergen [30]. Peri-screw radiolucency is a great deal more common than 3%. Classical, systemically detectable metal allergy therefore cannot account for most halos. The model proposed here does not require systemic sensitization, since it turns on local regulatory restraint rather than on a positive patch test. But the figure sets a ceiling on how much of the phenomenon any hypersensitivity mechanism could account for, and it recasts prediction 4 as a test of a minority effect rather than of the main one.

The second is the alloy. Two features of the hydroxyapatite series limit what can be carried forward from it. The rates quoted are screw-level, from 21 patients, and plasma-sprayed hydroxyapatite is no longer in routine spinal use. More seriously, the screws were stainless steel. Surgical stainless steel is specified at 13% to 15% nickel, and nickel is by some distance the most common implant allergen. The titanium alloy specification contains no nickel, and of the 118 patients patch-tested in the series above, not one reacted to titanium, aluminum, or vanadium [29]. Contemporary instrumentation is titanium alloy, so the model predicts that the change in alloy should have reduced halo rates substantially. We are not aware that it has. The only qualification available to us is a weak one: modern titanium systems may still contain trace nickel [30]. This is the strongest argument against the hypothesis in the clinical literature, and we cannot answer it.

The third is more awkward still. The systematic review above looked for radiological changes accompanying confirmed metalwork allergy and found almost none reported. Of the cases it assembled, one described incomplete arthrodesis at a single level. Another explicitly reported that there was no adjacent radiolucency beside the metalwork. The remainder, including the 796-patient cohort, made no comment on radiological changes at all [30]. If confirmed metal hypersensitivity in the spine were regularly accompanied by peri-implant osteolysis, that literature ought to show it. It does not. The absence may be one of reporting rather than the phenomenon itself, since none of those studies set out to examine bone, but it is an absence exactly where this model predicts a presence, and we record it as such.

Two series of failed, osteolytic joints report Tregs that are not reduced. One found them increased in periprosthetic tissue and blood [31]. The other found their number and suppressive capacity to be essentially normal [32]. Both are conference proceedings rather than full papers, but they are the only direct counts available. If the hypothesis were about numerical absence, they would be fatal to it. As framed here, it predicts functional rather than numerical failure: regulatory cells may be recruited in normal or increased numbers and still be disarmed by the local milieu. That reframing weakens the falsifiability of the hypothesis, and we acknowledge this. A mechanism is available for it: regulatory cells may be diverted toward a pro-inflammatory phenotype in an inflamed milieu while retaining Foxp3, in which case they would be counted and would not suppress. A Treg count at the interface would not test the model. A functional suppression assay would.

The conventional explanation for rheumatoid failure is poor bone quality, for which the evidence is direct, together with long-term corticosteroid use [17]. Neither is disputed here. It is also not obviously separable from the immunological explanation because the two derangements overlap. Separating them requires the covariate-adjusted design set out below, not further argument.

Many of these patients receive immunosuppression, which might be expected to quiet the effector response invoked here. A tempting answer is Sakaguchi's: a limited course preferentially removes activated regulatory cells [14]. It rests on murine work from an era of nonselective agents and does not transfer to current practice. Rheumatoid arthritis is now treated largely with TNF inhibitors, and anti-TNF antibody therapy restores the suppressive capacity of Tregs in these patients and induces a distinct regulatory population acting through TGF-β and interleukin-10 [33,34]. In the present model, that is a prediction rather than an embarrassment: patients with rheumatoid arthritis established on anti-TNF antibody therapy should develop halos less often than those who are not, which is prediction 10. Corticosteroids are a separate matter, since they impair osteoblast function in their own right.

The rheumatological corollary carries a further difficulty. Patients with rheumatoid arthritis have an increased risk of implant-associated infection because of both the disease and its treatment, and infection is among the outcomes reported to be increased in the pooled analysis [28]. Read alongside the sonication data, the whole rheumatoid excess could be an infection signal and nothing more. Any test of this corollary must therefore apply the composite infection workup to both arms, and not only to the cases nominated for the immune mechanism.

A simpler explanation of the terminal step deserves to be stated because it occurs in the same tissue as the hypothesis. Marrow adipocytes are not inert. Marrow adipose tissue increases with age and with falling bone density, and vertebral marrow fat fraction is an independent contributor to circulating soluble RANKL [20]. An adipocyte-derived rise in the RANKL-to-OPG ratio would produce peri-screw resorption in the same patients and at the same levels, with no T cell taking part, and on fewer assumptions than the model proposed here. The two accounts are separable. An adipocyte-driven process predicts that halo formation tracks marrow fat fraction; the present model predicts that it tracks regulatory function independently of fat fraction. Prediction 9 sets out the comparison.

Nothing above displaces the mechanical account or the conventional view in which peri-implant osteoclastogenesis pools innate macrophage signals with adaptive ones [5]. The likely relationship is synergistic: micromotion liberates ions and injures tissue, while immune-mediated resorption would increase micromotion in turn. Any immunological account must be additive, and the informative comparison for any test is the haloed screw against the loose but unhaloed screw, not against the well-fixed one.

Testable predictions

The hypothesis is worth holding only because it can be refuted. Table 3 sets out the predictions, the existing methods that would test each, and the results that would count against the model. The citations identify the methods, not confirmation of the predictions themselves. Four matter most. Prediction 1 is the one that would bring human tissue to bear, and only sampling stands in the way; being cross-sectional, it could establish association but not that the immune change preceded the bone loss. Prediction 6 is the decisive loss-of-function test. Prediction 8 must be satisfied before any of the others can be interpreted because it separates the proposed lesion from low-grade infection, and it can be applied in full only when the construct is revised. Prediction 11 is the least expensive of all, since it requires no new tissue, no animals, and no new imaging.

**Table 3:** Testable predictions of the local regulatory failure model, with the method that would test each and the observation that would refute it Citations indicate methods that already exist and could be applied to the question. They are not evidence for the prediction itself. BAC: bacterial artificial chromosome; CT: computed tomography; MRI: magnetic resonance imaging; OPG: osteoprotegerin; PCR: polymerase chain reaction; RANK: receptor activator of nuclear factor-κB; RANKL: RANK ligand; TNF: tumor necrosis factor; Treg: regulatory T-cell; CD4+: cluster of differentiation 4 positive; Foxp3+: forkhead box P3-positive

#	Prediction	How it could be tested (the method already exists)	Refuted if
1	Marrow around a haloed screw contains activated, metal-reactive CD4+ effector cells, with Foxp3+ Tregs depleted or functionally disarmed	Marrow sampled at revision from the former implant surface, with flow cytometry and immunohistochemistry, as previously applied to rheumatoid marrow at arthroplasty [27]	The infiltrate is neutrophil-dominant, or Foxp3+ cells are present and demonstrate normal suppressive function on functional assay
2	Patients with rheumatoid and related diseases develop halos more often, with wider zones and earlier onset, than matched degenerative controls	Prospective cohort study with CT-based bone density and recorded corticosteroid exposure entered as covariates [1,2,17]	The excess disappears after adjustment for bone density and corticosteroid exposure
3	Preoperative Treg suppressive function, rather than frequency, varies inversely with the subsequent probability of halo formation	Prospective cohort study with a preoperative functional suppression assay on circulating cells, alongside phenotyping, and protocol CT at fixed intervals [22,23]	Suppressive function and halo incidence prove unrelated. A frequency count alone would not test the model and is not proposed
4	A positive preoperative lymphocyte transformation test for the relevant metal flags patients who develop halo earlier and more extensively	Preoperative lymphocyte transformation testing, as used in arthroplasty hypersensitivity work, with blinded radiological follow-up [7]	Halo incidence is the same in test-positive and test-negative patients
5	The haloed interface carries an elevated RANKL-to-OPG ratio, with reduced local interleukin-10 and reduced available interleukin-2	Assay of interface tissue and fluid at revision against the RANKL-RANK-OPG axis [21,25]	The ratio is unshifted, or interleukin-10 is preserved
6	Reinforcing local Tregs lowers interface RANKL and blunts the radiological bone loss; depleting them accelerates and increases bone loss	Adoptive transfer or low-dose interleukin-2 at the screw in an instrumented animal. For depletion, transient and partial ablation is required: complete and continuous ablation of Foxp3+ cells produces a fatal scurfy-like syndrome long before a peri-screw endpoint can develop. Therefore, a BAC-transgenic line permitting reversible depletion or local intraosseous depletion must be used instead [14,35]	Transient Treg depletion leaves the peri-screw radiolucency unchanged. Note that systemic depletion confounds a local claim, which is a further argument for the intraosseous approach
7	Sealing the metal from the marrow suppresses the halo formation independently of anchorage	Comparison of surface treatments that seal without improving osseointegration, or of alloys matched for pull-out strength but differing in the allergenicity of the ions they shed [3]	Halo rates track fixation strength alone
8	Haloed screws attributed to this mechanism are culture- and sonication-negative and do not respond to antimicrobial therapy	Composite infection workup at revision: inflammatory markers, multiple prolonged tissue cultures, implant sonication, molecular (PCR) testing, and histopathology [18]. This workup is available in full only in operative cases; where the construct is not revised, infection can be assessed only indirectly using inflammatory markers and the clinical course	Sonication or extended culture recovers organisms at rates comparable to those in unselected loose screws, or the lesion resolves with antibiotics
9	Halo formation tracks regulatory function independently of vertebral marrow fat fraction	Chemical shift MRI for marrow proton density fat fraction at the instrumented levels, entered alongside a functional regulatory assay in the same prospective cohort [20]	Halo formation tracks marrow fat fraction and adds nothing once fat fraction is accounted for, which would favor an adipocyte-derived RANKL source over the model proposed here
10	Rheumatoid patients receiving established anti-TNF antibody therapy develop fewer halos than rheumatoid patients who are not receiving anti-TNF therapy	Drug-class stratification within the proposed cohort, with current biologic and disease-modifying therapy recorded prospectively [33,34]	Halo incidence is the same across drug classes or is higher in the anti-TNF group
11	The distribution of the halo across instrumented levels is flatter than the distribution of mechanical loosening	Level-by-level analysis of halo and of loosening in existing instrumented series; no new tissue, animal, or imaging work required [1,2]	Halo distribution matches the mechanical distribution, with halos concentrating at S1, the caudal-most instrumented level and the junctional levels

Tissue for prediction 1 would be obtained at revision from the cancellous bone directly abutting the former implant surface, before irrigation and at a defined distance from the interface, with a paired control sample from a noninstrumented level in the same patient. Without that paired control, any lymphocytic infiltrate could be dismissed as a property of that patient's marrow. Samples would be characterized by flow cytometry and immunohistochemistry, with the primary readout being a functional suppression assay rather than a cell count.

Proposed study design and sample size

Prediction 2 is the one a clinical group could act on now, and Table 3 states it too briefly to be implemented as written. The design would be a multicenter prospective cohort of patients with rheumatoid arthritis compared with matched degenerative controls, multicenter by necessity, since few units see many such patients undergoing instrumented fusion. Entry criteria should be narrow, since axial spondyloarthritis behaves in the opposite direction at the bone, and enrollment should be restricted to a single screw trajectory and fixation type. Matching would include age, sex, levels instrumented, alloy, and the two confounders that would otherwise drive the result: bone mineral density from the perioperative CT and cumulative corticosteroid exposure [17].

The primary outcome would be defined per patient: at least one screw with a circumferential radiolucency wider than 1 mm on protocol CT at fixed intervals over two years, acquired using a single prespecified CT protocol with metal artifact reduction and read on bone windows orthogonal to each screw axis by two blinded observers, with adjudication and interobserver agreement reported. Time to halo and maximal zone width would be secondary outcomes. That reading is morphological and inherits the reliability problem described above, so a quantitative endpoint should run alongside it: mean attenuation in a standardized peri-screw region of interest, measured from the same scan using the technique already used for vertebral bone density [17]. A fall confined to that region, with vertebral body attenuation preserved, would distinguish local resorption from generalized bone loss.

Infection must be excluded prospectively rather than argued away during analysis, but how completely it can be excluded depends on whether the patient undergoes surgery. In operative cases, the composite workup of prediction 8 applies in full and identically in both arms: peri-implant tissue samples held in extended culture for at least 14 days, sonication fluid culture of the explanted screw, broad-range molecular testing, and histopathology. The 14 days matter: the organisms recovered from loose but clinically quiet spinal implants are low-virulence skin commensals that routine short-incubation culture misses [18]. When a halo is found on protocol CT in a patient who is not revised, none of that tissue is available. Exclusion then rests on inflammatory markers and the clinical course and should be recorded as the weaker exclusion that it is: such a screw counts in the radiological endpoint, but it cannot be designated an immune lesion.

Fusion status must be assessed from the same scans and entered as both a stratification factor and a covariate, since a screw in a fused construct is largely unloaded, whereas one spanning a pseudarthrosis is subjected to repeated loading; without this adjustment, a positive result in the rheumatoid arthritis arm could be explained by a higher nonunion rate alone. Protocol imaging is a research exposure, and the cumulative radiation dose should be documented in the consent materials.

The numbers carry their assumptions with them. We assume a halo incidence of 20% among controls at two years, which is an assumption rather than an established figure, since the literature reports zone frequencies per screw more often than per patient. The target difference is the lower bound of the two- to fourfold excess reported for rheumatoid spines, that is, 40% versus 20% [28]. That excess was measured for reoperation and complications rather than for the halo, so the borrowing is an assumption on top of an assumption. A two-sided test of two independent proportions at α=0.05 with equal allocation then requires 82 patients per arm for 80% power and 109 for 90%, before allowance for continuity correction or attrition; 30% versus 20% would require about 294 per arm, beyond most consortia. The arithmetic behind these figures belongs in a trial protocol rather than here.

The unit of analysis needs careful consideration, since the outcome is per patient and the observation is per screw. The primary population would be all enrolled patients with at least one interpretable CT, with screw-level analyses being secondary and clustering accounted for, survival methods used for time to halo, and an ordinal model used for maximal width. Bone mineral density, corticosteroid dose, fusion status, construct and alloy, disease activity and duration, seropositivity, and current drug class would be prespecified as adjustment variables, the last being needed in any case to test the anti-TNF prediction. We would regard the clinical corollary as having failed if such a cohort returned an adjusted rate ratio whose confidence interval included unity. The calculations rest on an assumed control incidence, and statistical review would be appropriate before the study is conducted.

Limitations and alternative explanations

The main limitation is simple. No one has shown, in human vertebral marrow, either in situ recognition of metal or Treg failure at a haloed screw. No retrieval series from revised spinal instrumentation has characterized the peri-screw infiltrate by immunophenotype. Peri-screw tissue has been examined histologically: the sheep arm of the defining study found bone-implant contact reduced to 8% beneath a zone versus 55% where there was none [3]. That work quantified how much bone had been lost, not which cells removed it. The tissue-level evidence is otherwise almost entirely derived from arthroplasty synovium and the periprosthetic membrane [19,26,31,32]. The model has no direct human spinal support and should be read accordingly. The same absence means prediction 1 is untested rather than merely unconfirmed, so a single well-designed retrieval study would weigh heavily for or against the model. It could not settle it. A retrieval series samples one moment, after the halo has formed, and can show association but not the order of events. The reliability of the radiological endpoint remains a further limitation of any such test [16].

There is a tension the model must live with. Systemic bone mineral density tracks halo formation poorly, yet osteoporotic and rheumatoid patients experience loosening more often. The cleanest reconciliation treats systemic bone mineral density as a background liability and local events as the proximate driver [17]. That is a reconciliation, not a finding.

Orthopedic implant hypersensitivity is a recognized entity with an established diagnostic framework, yet no patient with rheumatoid arthritis undergoing spinal instrumentation has been documented as having hardware removed for confirmed metal hypersensitivity, supported by patch or lymphocyte transformation testing and resolution after explantation [6]. The lymphocyte transformation test itself is poorly standardized, and any diagnostic weight placed on it must be qualified. Skin patch testing raises the same difficulty from the other side. In the pediatric cohort above, laboratory hypersensitivity did not correlate with postoperative allergic reactions, and a cutaneous response may not represent the response to an indwelling implant [29].

One alternative must be distinguished from the hypothesis at the bedside. Low-grade implant infection must be excluded through a composite assessment that may include inflammatory markers, multiple prolonged tissue cultures, sonication where feasible, molecular testing, and histopathology. That assessment can be performed only in patients who undergo revision. When the construct remains in place, exclusion rests on inflammatory markers and the clinical course and is correspondingly weaker. Normal inflammatory markers or negative routine cultures do not reliably exclude biofilm-associated infection [18]. The lesion proposed here should be culture- and sonication-negative, lymphocyte-predominant rather than neutrophil-predominant, associated with metal sensitivity on lymphocyte transformation testing, and unresponsive to antibiotics. Making that distinction prospectively is among the most achievable tests of the model.

## Conclusions

A pedicle screw differs from an arthroplasty implant in one respect that may be immunologically relevant. Part of its surface lies within the bone marrow, where it is in contact with resident lymphocytes capable of mounting a type IV response. Whether such a response occurs there and whether loss of local regulatory restraint could lead to the bone loss seen as a halo remain unknown.

Patients with rheumatoid arthritis and related diseases undergo more reoperations and experience more implant-related complications, although an increased incidence of the halo itself has not been demonstrated. Whether that excess reflects the mechanism proposed here, the poor bone quality and corticosteroid exposure usually invoked, or indolent low-grade infection is exactly what remains to be tested. What can honestly be claimed is narrower than the title suggests. The value of the framework therefore lies less in the label than in the experiments it makes obligatory: an immunophenotyped retrieval series, a covariate-adjusted prospective cohort, and a depletion-and-reconstitution experiment in an instrumented animal model. Until those studies are completed, this remains a hypothesis and nothing more.
